# Surgery

**Published:** 1905-12

**Authors:** James Swain


### SURGERY.

**The Surgical Treatment of hæmatemesis from gastric ulcer** is still the subject of various opinions, and we are indebted to Dr. Gilman Thompson [1] for an exhaustive inquiry into over 200 cases, with a view to determining the indications for operative treatment in this condition.

It is interesting to note in passing that in none of the cases has the study of the chemical analysis of gastric contents proved of determining value in the diagnosis, for the results have been too variable, not rarely in the same patient on successive days varying from acidity to hyperacidity. Additional difficulty is encountered in the variability and inconstancy of all the so-called "cardinal symptoms," such as pain, vomiting, or tenderness, any one of which may spontaneously disappear or give place to another.

He is not yet prepared to accept a definite clinical classification of gastric ulcer into acute and chronic types based upon hæmatemesis, as attempted by Moynihan, Samuel and others. This classification is based upon the fact that a number of cases occur in which there is a sudden single hemorrhage, followed by complete and permanent recovery under medical treatment, whereas with chronic ulcers there may be repeated small or large hemorrhages and other gastric symptoms recurring at intervals through months or years. Dr. Thompson, on the other hand, considers that a single large hemorrhage may denote a chronic ulcer, equally with an acute one.

To estimate the mortality of gastric ulcer by statistical methods is difficult, but of 195 cases with hæmatemesis received

[1] *Am. J. M. Sc.*, 1905, cxxx. 375.

into the Presbyterian Hospital 41 were fatal, and if to this be added the increased mortality arising from those cases which had not run their course, there is good ground for the plea of earlier surgical intervention in fairly typical cases. The case in favour of operation is made strong because the mortality of gastro-enterostomy for gastric ulcer appears to be gradually lessening under improved technique. Thus we find that Mayo Robson's [1] mortality rate for his own cases is only five per cent., and Moynihan's [2] figures are 153 gastro-enterostomies for ulcer with only two deaths, but in 22 cases of gastric hemorrhage which apparently would have soon proved fatal there were 19 recoveries after operation.

Dr. Blake [3] considers that a single large hemorrhage, without previous symptoms referable to ulcer, should not be operated upon, but when there have been antecedent symptoms operation should be performed. He also considers that cases suffering from recurrent hemorrhage should be subjected to operation. In these views Dr. Thompson concurs, and he would add that those chronic cases also should be operated upon in which the patient's life is either made miserable through pain, or endangered through persistent vomiting and emaciation, provided medical treatment tried for two or three weeks has failed to produce any improvement. This last indication has not yet met with the general acceptance which it deserves, but the writer of this article can bear testimony to the soundness of the view held, and to the excellent result of operative treatment in such cases.

Let the surgeon be given the advantage of dealing with cases not already made hopeless through anæmia, ulcers on the verge of perforation, or complicated by a mass of perigastric adhesions. To offset these grave dangers, an early gastro-enterostomy offers freedom from further hemorrhage, from chronic hyperacidity, pain, traumatic irritation of the ulcer by food, motor insufficiency, "hour-glass" stomach, and gastric dilatation.

**Fracture of the Carpal Scaphoid and Dislocation of the Semi-lunar Bone.**—A sprained wrist is generally regarded as fairly easy of diagnosis and treatment, but Codman and Chase [4] maintain that many of these cases are examples of fracture or dislocation of the carpal bones, and they rightly consider that these injuries should be as definitely recognised as those of fracture or dislocation of any other bone. They show that in the large majority of sprains of the wrist which do not promptly recover the injury consists either in a simple fracture of the scaphoid, or an anterior dislocation of the semi-lunar bone. These two injuries are frequently combined, and in such cases

[1] *Phila. M. J.*, 1901, vii. 1005. [2] *Brit. M. J.*, 1905, i. 753.
[3] *Am. J. M Sc.*, 1904, cxxviii. 985. [4] *Ann. Surg.*, 1905, xli. 321, 863.

the proximal fragment of the scaphoid is usually dislocated forward with the semi-lunar bone.

Simple fracture of the scaphoid gives a definite clinical picture, and may be recognised, even without the X-rays, by the association of the following symptoms:—(*a*) The history of a fall on the extended hand; (*b*) Localised swelling in the radial half of the wrist-joint; (*c*) Acute tenderness in the anatomical "snuff-box" when the hand is adducted; (*d*) Limitation of extension by muscular spasm, the overcoming of which by force causes unbearable pain.

It appears that a broken scaphoid has little power of repair, and is capable of but little callus formation; and, therefore, those cases which remain untreated or are treated by massage and active and passive motion generally, if not always, remain ununited, and the original symptoms persist for years with only slightly abated intensity. On the other hand, cases of fracture of the scaphoid may unite if motion of the wrist is prevented during the first four weeks after injury, but if by this time no union has occurred future union is unlikely.

The authors consider that excision of the proximal half of a fractured scaphoid gives a somewhat better result than conservative treatment. This operation is best performed through a posterior incision to the outer side of the tendons of the extensor communis digitorum. Passive motion of the wrist joint and active movements of the fingers should be commenced within a week after the operation. (The possibility of a bipartite scaphoid should be considered in interpreting skiagrams of simple fractures of the scaphoid, but its occurrence must be very rare in comparison with fracture.)

The other injury referred to—anterior dislocation of the semi-lunar bone—should be recognised clinically, even without the X-rays, by the association of the following symptoms:—(*a*) The history of an injury of considerable violence to the extended or twisted wrist; (*b*) A "silver-fork" deformity, the posterior prominence of which corresponds with the head of the os magnum, and between which and the lower end of the radius is found a groove representing the position formerly occupied by the now anteriorly dislocated semi-lunar bone; (*c*) A swelling under the flexor tendons of the wrist just anterior to the lower end of the radius; (*d*) A shortened appearance of the palm as compared with the other hand; (*e*) Stiffness of the partially flexed fingers, motion of which, either active or passive, is painful; (*f*) The persistence of the normal relations of the styloid processes of the radius and ulna, and the existence of the shortening of the distance from the radial styloid to the base of the first metacarpal.

As regards treatment of this condition, it has been found that recent dislocations of the semi-lunar bone may be reduced with good result, even after the fifth week, by hyperextension followed by hyperflexion over the thumbs of an assistant held firmly in the flexures of the wrist on the semi-lunar bone. Irreducible dislocations, however, demand excision of the semi-lunar bone and the whole or a portion of the scaphoid, if there is a coincident fracture of the latter.

**Carcinoma of the prostate** occurs in about 10 per cent. of the cases of prostatic enlargement, but it is only lately that this serious affection has become amenable to radical treatment. Young,[1] after the study of 40 cases, has devised an operation which he has successfully applied in four cases. An early diagnosis is essential, and it must be therefore remembered that the disease may begin as an isolated nodule in an otherwise benign hypertrophy, or a prostatic enlargement which has for many years furnished the symptoms and signs of benign hypertrophy may suddenly become evidently malignant. Marked induration, if only an intralobular nodule in one or both lobes of the prostate in men past fifty years of age, should be viewed with suspicion, especially if the cystoscope shows little intravesical prostatic outgrowth, and pain and tenderness are present.

The posterior surface of the prostate should be exposed as for an ordinary perineal prostatectomy, and if the operator is unable to make a positive diagnosis of malignancy, a longitudinal incision should be made on each side of the urethra (as in prostatectomy) and a piece of the tissue excised for frozen sections, which can be prepared in about six minutes and examined by the operator at once. If the disease is malignant the incisions may be cauterised and closed, and the radical operation performed.

Cancer of the prostate remains for a long time within the confines of the lobes, the urethra, bladder, and especially the posterior capsule of the prostate remaining unaffected for a considerable period. Extra-prostatic invasion nearly always occurs first along the ejaculatory ducts into the space immediately above the prostate between the seminal vesicles and the bladder, and beneath the fascia of Denonvilliers. Thence the disease gradually invades the inferior surface of the trigone and the lymphatics leading towards the lateral walls of the pelvis, but involvement of the pelvic glands occurs late, and often the disease gives rise to metastatic deposits in the osseous system without first invading the glands.

Cure can be expected only by radical measures, and the routine removal of the seminal vesicles, vasa deferentia, and most of the vesical trigone with the entire prostate. The opening in the bladder is closed after the anterior part has been stitched on to the divided urethra.

The four cases in which the author has performed this operation show the simplicity, effectiveness, and the remarkably satisfactory functional results of the procedure.

JAMES SWAIN, M.D., M.S. Lond.

[1] *Johns Hopkins Hosp. Bull.*, 1905, xvi. 315.

